# Age-related differences in breast cancer mortality according to race/ethnicity, insurance, and socioeconomic status

**DOI:** 10.1186/s12885-020-6696-8

**Published:** 2020-03-17

**Authors:** Yazmin San Miguel, Scarlett Lin Gomez, James D. Murphy, Richard B. Schwab, Corinne McDaniels-Davidson, Alison J. Canchola, Alfredo A. Molinolo, Jesse N. Nodora, Maria Elena Martinez

**Affiliations:** 1 Moores Cancer Center, University of California San Diego, La Jolla, CA USA; 2 Department of Epidemiology and Biostatistics, University of California San Francisco, San Francisco, CA USA; 3 Helen Diller Family Comprehensive Cancer Center, University of California San Francisco, San Francisco, CA USA; 4 San Diego State University Institute for Public Health, San Diego, CA USA; 5 Department of Family Medicine and Public Health, University of California San Diego, La Jolla, CA USA

**Keywords:** Mortality, Younger and older age, Breast cancer

## Abstract

**Background:**

We assessed breast cancer mortality in older versus younger women according to race/ethnicity, neighborhood socioeconomic status (nSES), and health insurance status.

**Methods:**

The study included female breast cancer cases 18 years of age and older, diagnosed between 2005 and 2015 in the California Cancer Registry. Multivariable Cox proportional hazards modeling was used to generate hazard ratios (HR) of breast cancer specific deaths and 95% confidence intervals (CI) for older (60+ years) versus younger (< 60 years) patients separately by race/ethnicity, nSES, and health insurance status.

**Results:**

Risk of dying from breast cancer was higher in older than younger patients after multivariable adjustment, which varied in magnitude by race/ethnicity (*P*-interaction< 0.0001). Comparing older to younger patients, higher mortality differences were shown for non-Hispanic White (HR = 1.43; 95% CI, 1.36–1.51) and Hispanic women (HR = 1.37; 95% CI, 1.26–1.50) and lower differences for non-Hispanic Blacks (HR = 1.17; 95% CI, 1.04–1.31) and Asians/Pacific Islanders (HR = 1.15; 95% CI, 1.02–1.31). HRs comparing older to younger patients varied by insurance status (*P*-interaction< 0.0001), with largest mortality differences observed for privately insured women (HR = 1.51; 95% CI, 1.43–1.59) and lowest in Medicaid/military/other public insurance (HR = 1.18; 95% CI, 1.10–1.26). No age differences were shown for uninsured women. HRs comparing older to younger patients were similar across nSES strata.

**Conclusion:**

Our results provide evidence for the continued disparity in Black-White breast cancer mortality, which is magnified in younger women. Moreover, insurance status continues to play a role in breast cancer mortality, with uninsured women having the highest risk for breast cancer death, regardless of age.

## Background

According to American Cancer Society, the 10-year probability of developing breast cancer increases with age, from 0.5% in women 30 years of age to 3.9% in those age 70 and the median age of diagnosis is 62 [1]. In 2015, 58.0% of all incident breast cancers in the United States (U.S.) occurred in women over the age of 60 [2]. With a rising number of older women in the U.S., understanding the breast cancer burden, including survival outcomes in these women is important. While different age cut-offs are used to define younger versus older patients, it is well recognized that women less than 40 years of age are more likely to develop breast cancer with more aggressive subtype and worse clinicopathological features [1, 3]. Findings from published studies report differences in breast cancer mortality and survival by age, showing that women diagnosed with breast cancer at less than 40 years of age have a lower survival than older patients [4–6]. Studies focused on breast cancer mortality have also reported that younger compared to older breast cancer patients have higher breast cancer mortality, regardless of age-cut off [7, 8]. While a wealth of published data exists on factors that impact breast cancer mortality, including race/ethnicity, socioeconomic status, and other sociodemographic factors [9–11], limited research exists on whether associations vary by age at diagnosis.

Few studies have been published on factors associated with breast cancer mortality in older women when compared to younger patients, with varying age cut-offs [12–17]. Published results show that older women with worse breast cancer health outcomes are more likely to be racial/ethnic minority women, have a lower sociodemographic status, and have no health insurance [13, 15, 17]. Under-treatment and/or more comorbidities in older women compared to younger women could be reasons for the observed higher breast cancer mortality in these women [12, 13, 16]. It has been reported that as women age, they are less likely to pursue or be offered aggressive treatment [12]. To our knowledge, studies of age differences in survival have not considered potential heterogeneity by sociodemographic factors, which would aid in better understanding breast cancer outcomes.

Using data from the population-based California Cancer Registry (CCR), our study assessed breast cancer mortality differences between younger (age 18–59) and older (age 60 and above) breast cancer patients according to race/ethnicity, health insurance, and socioeconomic status, while controlling for patient and clinical variables.

## Methods

### Study population

We obtained information from the CCR for female California residents ages 18 years and older at diagnosis, who were diagnosed with a first, primary invasive breast cancer [International Classification of Disease for Oncology, 3rd Edition, (ICD-O-3) site codes C50.0–50.9] during January 1, 2005 through December 31, 2015 (*n* = 219,266). Patients were excluded from the analysis hierarchically as follows: diagnosis by death certificate or autopsy only (*n* = 889) or diagnosis not microscopically confirmed (*n* = 1698); ICD-O-3 histologic type other than: 8000, 8001, 8010, 8020, 8022, 8050, 8140, 8201, 8211, 8230, 8255, 8260, 8401, 8453, 8480, 8481, 8500–8525, or 8575 (*n* = 3654); tumor size missing because unknown (*n* = 8347), no tumor noted (*n* = 510), microscopic (*n* = 2250), diffuse (*n* = 608), or mammographic diagnosis only (*n* = 59); age < 60 insured by Medicare (*n* = 513); no follow-up (*n* = 269); residential address that was uncertain or not geocodable (*n* = 6292). The study included 192,932 patients, of whom 94,076 were younger (age 18–59) and 98,856 were older (age 60 and above, up to age 109) patients.

### Data acquisition

Data from the CCR, mostly derived from the patient’s medical record, were used to obtain age at diagnosis, marital status, residential address at diagnosis, stage at diagnosis, tumor size (in centimeters), lymph node involvement, histology, grade (I, II, III/IV, or unknown), and hormone receptor [estrogen receptor (ER), progesterone receptor (PR), and human epidermal growth factor receptor 2 (HER2)] status. The CCR followed patients for vital status, from linkage with vital records, to December 31, 2015 for this study.

For the variables of interest in the present report, we used data from the medical record to classify race/ethnicity as non-Hispanic White (NHW), non-Hispanic Black, Hispanic, Asian/Pacific Islander (API), or other/unknown; and primary and secondary source of payment were used to classify insurance status, as private only, Medicare only/Medicare + private, any Medicaid/military/other public, no insurance, and unknown; in the CCR, payer status is coded based on the most extensive insurance type across the diagnosis to treatment continuum. We used a multi-component measure of neighborhood socioeconomic (nSES), based on patients’ residential census block group at diagnosis. This measure incorporated the 2000 U.S. Census (for cases diagnosed in 2005) and the 2006–2010 American Community Survey data (for cases diagnosed in 2006 and forward) on education, occupation, unemployment, household income, poverty, rent, and house values [18, 19]. Each patient was assigned a nSES quintile, based on the distribution of socioeconomic status across census block groups in California.

### Statistical analysis

Given the lack of standard for categorizing younger and older breast cancer patients, we used the median age of the study population as a cut-off (60 years); younger women included those age 18–59 and older included 60+ years. Differences in mortality for older and younger women were examined by two methods. First, comparisons were made between older and younger patients stratified by race/ethnicity, insurance status, and nSES, with younger women as the reference group. Next, models were stratified by age and comparisons were made between race/ethnicity (non-Hispanic White as the reference group), insurance status (private insurance as the reference group), and nSES quintiles (5th quintile as the reference group).

Descriptive statistics were calculated and reported as percentages for categorical data and means with standard deviation for continuous variables. Covariates were shown overall and for younger and older women. Covariates examined included: age (continuous and categorical), race/ethnicity, marital status, insurance status, nSES, whether the patient was seen at one or more of the National Cancer Institute-designated Cancer Centers in California (NCICC) for her breast cancer, American Joint Committee on Cancer (AJCC) stage at diagnosis, tumor subtype, lymph node involvement, tumor size, tumor grade, and tumor histology.

Follow-up time was calculated as the number of days between the date of diagnosis and date of death from breast cancer (ICD 9/10 = 174/C50), the date of death from another cause, the date of last follow-up (i.e., last known contact), or the study end date (12/31/2015). There were 599 deceased patients with an unknown cause of death which were excluded from all models. Cox proportional hazards regression was used to estimate breast cancer specific hazard rate ratios (HR) and corresponding 95% confidence intervals (CI). Adjusted models were stratified by AJCC stage and adjusted for age at diagnosis (continuous), year of diagnosis (continuous), race/ethnicity, marital status, insurance status, nSES, whether the patient was seen at one or more of the NCICC in California for her breast cancer, tumor subtype, lymph node involvement, tumor size, tumor grade, and tumor histology. Fully adjusted models were additionally adjusted for clustering by block group, using a sandwich estimator of the covariance structure that accounts for intracluster dependence. The proportional hazards assumption was tested by examining the correlation between time and scaled Schoenfeld residuals for all covariates. The proportional hazards assumption was violated for AJCC stage at diagnosis, tumor subtype, and tumor grade. Stage was included as an underlying stratifying variable in the fully adjusted Cox regression models reported here, which allowed the baseline hazards to vary by stage. Additionally, stratifying the Cox model by tumor subtype and tumor grade did not meaningfully change the HR for the main effect of age, so these factors were simply adjusted for in fully adjusted models. Wald Type 3 tests for interaction between age group (18–59, 60+) and race/ethnicity, insurance status, and nSES and were computed using cross-product terms, in models adjusted for all statistically significant (*p* < 0.05) interactions with age group (race/ethnicity, marital status, insurance status, NCICC, tumor subtype, and lymph node involvement). Wald tests for trend across nSES quintiles were computed using quintile number as an ordinal variable. All statistical tests were carried out using SAS software version 9.3 (SAS Institute).

## Results

Of the total population (*n* = 192,932), 94,076 (48.7%) were diagnosed under the age 60 and 98,856 (51.2%) were aged 60 and older. As noted in Table 1, approximately 60% of the population was NHW, 40% was married, 26% was from the highest socioeconomic neighborhood, and 58% had private insurance. Examining clinical factors, 48% of total patients were diagnosed with stage I breast cancer, 65% had hormone receptor-positive (ER positive or PR positive)/HER2-negative tumor subtype, 66% were negative for lymph node involvement, 35% had a tumor size of 1–2 cm, 42% had a tumor grade of II, 79% had a ductal histology, and 12% received care at a NCI-designated Cancer Center.

**Table 1:** Patient demographic and clinical characteristics for younger (18–59 years) and older (60+ years) age at breast cancer diagnosis, California, 2005–2015

	All		Younger (18–59)		Older (60+)	
**Total Number of Patients**	192,932	100.0	94,076	100.0	98,856	100.0
**Age (yrs) Mean (SD)**	60.2(13.7)		48.8(7.3)		71.2(8.3)	
**Age category**						
18–39	10,785	5.6	10,785	11.5	–	–
40–49	35,297	18.3	35,297	37.5	–	–
50–59	47,994	24.9	47,994	51.0	–	–
60–69	49,225	25.5	–	–	49,225	49.8
70–79	31,540	16.3	–	–	31,540	31.9
80+	18,091	9.4	–	–	18,091	18.3
**Race/ethnicity**						
Non-Hispanic White	116,534	60.4	49,107	52.2	67,427	68.2
Non-Hispanic Black	12,014	6.2	6348	6.7	5666	5.7
Hispanic	36,363	18.8	22,305	23.7	14,058	14.2
Asian/Pacific Islander	25,871	13.4	15,242	16.2	10,629	10.8
Other/unknown	2150	1.1	1074	1.1	1076	1.1
**Marital status**						
Married	77,754	40.3	31,324	33.3	46,430	47.0
Unmarried	107,808	55.9	59,344	63.1	48,464	49.0
Unknown	7370	3.8	3408	3.6	3962	4.0
**Neighborhood (block group) statewide SES quintile**						
1st (lowest)	23,702	12.3	12,174	12.9	11,528	11.7
2nd	33,174	17.2	15,787	16.8	17,387	17.6
3rd	39,136	20.3	18,584	19.8	20,552	20.8
4th	45,882	23.8	22,231	23.6	23,651	23.9
5th (highest)	51,038	26.5	25,300	26.9	25,738	26.0
**Insurance status**						
Private only	112,154	58.1	71,611	76.1	40,543	41.0
Medicare only or Medicare+Private	40,789	21.1	-^a^	-^a^	40,789	41.3
Any Medicaid/Military/Other public	33,225	17.2	18,671	19.8	14,554	14.7
No insurance	1598	0.8	1085	1.2	513	0.5
Unknown	5166	2.7	2709	2.9	2457	2.5
**National Cancer Institute-–designated cancer center**						
No	170,509	88.4	80,161	85.2	90,348	91.4
Yes	22,423	11.6	13,915	14.8	8508	8.6
**AJCC Stage**						
I	91,898	47.6	39,390	41.9	52,508	53.1
II	68,825	35.7	36,785	39.1	32,040	32.4
III	22,492	11.7	13,251	14.1	9241	9.3
IV	7249	3.8	3609	3.8	3640	3.7
	2468	1.3	1041	1.1	1427	1.4
**Tumor subtype**						
ER+, PR+/HER2-	125,240	64.9	56,822	60.4	68,418	69.2
ER+, PR+//HER2+	19,630	10.2	11,808	12.6	7822	7.9
ER-, PR−//HER2+	9023	4.7	5467	5.8	3556	3.6
Triple negative	19,836	10.3	11,280	12.0	8556	8.7
Unclassified	19,203	10.0	8699	9.2	10,504	10.6
**Lymph node involvement**						
Negative	126,915	65.8	56,446	60.0	70,469	71.3
Positive	64,045	33.2	36,933	39.3	27,112	27.4
Unknown	1972	1.0	697	0.7	1275	1.3
**Tumor size (cm)**						
0.10 ≤ tumor ≤0.50	13,488	7.0	6275	6.7	7213	7.3
0.50 < tumor ≤1.00	31,342	16.2	12,706	13.5	18,636	18.9
1.00 < tumor ≤2.00	67,792	35.1	31,674	33.7	36,118	36.5
2.00 < tumor ≤5.00	64,694	33.5	34,472	36.6	30,222	30.6
> 5.00	15,616	8.1	8949	9.5	6667	6.7
**Grade**						
Grade I	43,518	22.6	17,726	18.8	25,792	26.1
Grade II	80,909	41.9	37,234	39.6	43,675	44.2
Grade III/IV	61,205	31.7	35,688	37.9	25,517	25.8
Unknown	7300	3.8	3428	3.6	3872	3.9
**Histology**						
Ductal	151,631	78.6	76,477	81.3	75,154	76.0
Lobular	31,554	16.4	13,564	14.4	17,990	18.2
Other	9747	5.1	4035	4.3	5712	5.8

^a^ Medicare-insured patients < 60 years of age were excluded

Table 2 shows the multivariable-adjusted breast cancer specific mortality for older versus younger women according to race/ethnicity, health insurance, and nSES. Overall, older breast cancer patients had a higher risk of dying from breast cancer than younger patients (HR = 1.35; 95% CI, 1.29–1.40). HRs (95% CIs) comparing older to younger women were: 1.43 (1.36–1.51) for NHWs; 1.37 (1.26–1.50) for Hispanics; 1.17 (1.04–1.31) for non-Hispanic Blacks; and 1.15 (1.02–1.31) for APIs. A higher risk of dying was shown for older vs. younger patients for women with private insurance (HR = 1.51; 95% CI, 1.43–1.59) and for those with any Medicaid/military/other public insurance (HR = 1.18; 95% CI, 1.10–1.26). No significant differences by age were shown for uninsured patients. HRs by age across nSES quintiles ranged from 1.30 (95% CI, 1.19–1.41) in the third quintile to 1.42 (95% CI, 1.29–1.56) in fifth quintile.

**Table 2:** Breast cancer specific hazard ratios comparing older to younger age at diagnosis, stratified by race/ethnicity, insurance status, and neighborhood (block group) statewide SES quintile, California, 2005–2015

	Younger (18–59) No. deaths due to breast cancer	Older (60+) No. deaths due to breast cancer	HR (95% CI)^**a**^ Older vs. Younger (referent)	HR (95% CI)^**b**^ Older vs. Younger (referent)
**Overall**	7058	7706	1.14 (1.10–1.18)	1.35 (1.29–1.40)
**Race/ethnicity**				
Non-Hispanic White	3309	5199	1.29 (1.24–1.35)	1.43 (1.36–1.51)
Non-Hispanic Black	926	682	0.91 (0.83–1.01)	1.17 (1.04–1.31)
Hispanic	1892	1158	1.03 (0.96–1.11)	1.37 (1.26–1.50)
Asian/Pacific Islander	873	628	1.15 (1.04–1.27)	1.15 (1.02–1.31)
Other/unknown	58	39	0.75 (0.50–1.13)	0.56 (0.32–0.97)
**Insurance status**^**c**^				
Private only	4258	2606	1.18 (1.12–1.24)	1.51 (1.43–1.59)
Any Medicaid/Military/Other public	2360	1585	0.87 (0.82–0.93)	1.18 (1.10–1.26)
No insurance	169	76	0.96 (0.74–1.26)	1.05 (0.78–1.43)
Unknown	271	174	0.79 (0.65–0.95)	1.18 (0.96–1.45)
**Neighborhood (block group) statewide SES quintile**				
1st (lowest)	1375	1228	1.01 (0.93–1.09)	1.35 (1.23–1.48)
2nd	1482	1595	1.07 (0.99–1.15)	1.24 (1.14–1.36)
3rd	1509	1649	1.09 (1.02–1.17)	1.30 (1.19–1.41)
4th	1451	1670	1.19 (1.11–1.28)	1.43 (1.31–1.56)
5th (highest)	1241	1564	1.37 (1.27–1.48)	1.42 (1.29–1.56)

*HR* Hazard ratio, *CI* Confidence interval, *No.* Number

^a^ Adjusted for year at diagnosis

^b^ Stratified by AJCC stage and adjusted for year of diagnosis, marital status, race/ethnicity (in models not stratified by this), insurance status (in models not stratified by this), nSES (in models not stratified by this), lymph node involvement, tumor subtype, tumor size, tumor grade, tumor histology, NCI-designated cancer center and clustering by block group

^c^ Medicare-insured patients < 60 years of age were excluded

Table 3 shows HRs stratified for older and younger women separately, according to race/ethnicity, insurance status, and nSES. Compared to NHWs, non-Hispanic Blacks had a higher risk of dying regardless of age group, with higher HRs for younger (HR = 1.36; 95% CI, 1.25–1.48) than older (HR = 1.11; 95% CI, 1.01–1.22) patients. API women had lower risk of dying compared to NHWs in both age groups: HR (95% CI) was 0.88 (0.82–0.95) in younger and 0.77 (0.70–0.84) in older patients. No mortality difference was observed for Hispanics compared to NHWs in either age group. In younger women, compared to patients with private health insurance, a higher risk of breast cancer mortality was observed in women with any Medicaid/military/other public insurance (HR = 1.49; 95% CI, 1.41–1.58) and in those with no insurance (HR = 1.96; 95% CI, 1.65–2.32). As noted in the Methods, younger patients with Medicare insurance were excluded from the analysis. A higher risk of mortality was shown among older women with any Medicaid/military/other public insurance (HR = 1.13; 95% CI, 1.06–1.21) and those with no insurance (HR = 1.57; 95% CI, 1.22–2.03), as compared to privately-insured patients. No difference was observed for Medicare only or Medicare plus private insurance in the older group. In both younger and older women, breast cancer mortality risk decreased with increasing nSES quintile (*P*-trend = < 0.0001 for younger and < 0.0001 for older patients).

**Table 3:** Breast cancer specific hazard ratios stratified by age for race/ethnicity, health insurance, and neighborhood socioeconomic status, California, 2005–2015

	Younger (18–59)			Older (60+)		
	No. deaths due to breast cancer	HR (95%CI)^**a**^	HR (95%CI)^**b**^	No. deaths due to breast cancer	HR (95%CI)^**a**^	HR (95%CI)^**b**^
**All**	7058			7706		
**Race/ethnicity**						
Non-Hispanic White	3309	1.00	1.00	5199	1.00	1.00
Non-Hispanic Black	926	2.36 (2.20–2.54)	1.36 (1.25–1.48)	682	1.79 (1.66–1.94)	1.11 (1.01–1.22)
Hispanic	1892	1.42 (1.34–1.50)	0.95 (0.89–1.01)	1158	1.26 (1.18–1.34)	0.95 (0.88–1.02)
Asian/Pacific Islander	873	0.90 (0.84–0.97)	0.88 (0.82–0.95)	628	0.89 (0.81–0.96)	0.77 (0.70–0.84)
Other/unknown	58	0.91 (0.70–1.18)	0.83 (0.64–1.08)	39	0.58 (0.42–0.79)	0.50 (0.35–0.70)
	*P-interaction = < 0.0001*^c^					
**Insurance status**						
Private only	4258	1.00	1.00	2606	1.00	1.00
Medicare only or Medicare+Private	-^d^	-^d^	-^d^	3265	1.04 (0.98–1.10)	1.00 (0.94–1.05)
Any Medicaid/Military/Other public	2360	2.60 (2.47–2.74)	1.49 (1.41–1.58)	1585	1.73 (1.62–1.84)	1.13 (1.06–1.21)
No insurance	169	3.04 (2.61–3.54)	1.96 (1.65–2.32)	76	2.74 (2.18–3.44)	1.57 (1.22–2.03)
Unknown	271	1.47 (1.30–1.66)	1.11 (0.98–1.27)	174	0.92 (0.79–1.08)	0.86 (0.74–1.01)
	*P-interaction = < 0.0001*^c^					
**Neighborhood (block group) statewide SES quintile**						
1st (lowest)	1375	2.65 (2.45–2.86)	1.34 (1.22–1.46)	1228	1.93 (1.79–2.08)	1.38 (1.27–1.50)
2nd	1482	2.09 (1.93–2.25)	1.34 (1.24–1.45)	1595	1.60 (1.49–1.71)	1.28 (1.18–1.38)
3rd	1509	1.73 (1.61–1.87)	1.27 (1.17–1.37)	1649	1.36 (1.27–1.45)	1.19 (1.10–1.27)
4th	1451	1.38 (1.28–1.49)	1.13 (1.05–1.22)	1670	1.18 (1.10–1.26)	1.12 (1.04–1.20)
5th (highest)	1241	1.00	1.00	1564	1.00	1.00
	*P-interaction = 0.48*^c^					

*HR* Hazard ratio, *CI* Confidence interval, *No.* Number

^a^ Adjusted for age at diagnosis and year at diagnosis

^b^ Stratified by AJCC stage and adjusted for age of diagnosis, year of diagnosis, marital status, race/ethnicity, insurance status, nSES, lymph node involvement, tumor subtype, tumor size, tumor grade, tumor histology, NCI-designated cancer center and clustering by block group

^c^*P* for interaction between age group (younger and older) and race/ethnicity, insurance status, or nSES from a model that included all significant interactions with age group

^d^ Medicare-insured patients < 60 years of age were excluded

Recognizing that very young breast cancer patients (< 40 years of age) are more likely to have aggressive tumor subtypes or to have germline mutations [20], resulting in higher mortality compared to older patients, we conducted analyses excluding women less than 40 years of age. The fully adjusted HR (95% CI) comparing women 60 years of age and older to those less than 60 years was 1.35 (1.30–1.41), indicating no difference in the magnitude of the association compared to our main analysis including younger women. Results of analyses for race/ethnicity, health insurance, and nSES stratified by age were not materially different after excluding women less than 40 years of age than those including these younger patients (data not shown).

## Discussion

To our knowledge, there are no published reports on differences in breast cancer mortality between older compared to younger women according to sociodemographic characteristics, such as race/ethnicity, insurance status, and nSES. As such, this study contributes to the limited literature, showing that breast cancer patients 60 years of age and older had higher breast cancer mortality risk compared to those less than 60 years, with largest differences seen among NHW and Hispanic women, and among women with private insurance.

Our analyses assessed age-related differences in breast cancer mortality using two approaches. The first involved examining mortality differences in older versus younger patients within racial/ethnic, insurance, and nSES groups. In the second approach, we assessed differences across race/ethnicity, insurance status, and nSES among younger and older patients. Results of the first approach showed differential age effects by race/ethnicity and insurance status, but not by nSES. Although older women were at higher risk of dying from breast cancer compared to younger women across all racial/ethnic groups, differences were smaller for non-Hispanic Black and API patients than for NHWs and Hispanics. For insurance status, age-related mortality differences were more pronounced among privately-insured patients and no differences were shown for uninsured women. Finally, in regard to nSES, mortality differences between older and younger women were not highly variable.

In the second approach, comparisons across race/ethnicity, insurance status, and nSES within younger and older age groups showed mortality risk patterns that differed between the two age groups. Compared to NHWs, non-Hispanic Black women had higher risk of dying regardless of age group, although the HR was higher in magnitude in younger than older patients. The opposite pattern was observed for APIs who had lower risk of dying compared to NHWs. These findings are consistent with the well-established Non-Hispanic Black-White [1, 21] and API-White [1] mortality disparities previously reported and confirms the consistency of these patterns among older and younger women. The more pronounced Black-White survival difference in younger than older patients may reflect more aggressive disease diagnosed among younger non-Hispanic Black women, and warrants further study [1, 22]. No difference in mortality was observed between Hispanics and NHWs in either age group. Finally, results for nSES showed that patients residing in lower socioeconomic neighborhoods had higher breast cancer mortality compared to those in higher socioeconomic neighborhoods, regardless of age.

Differences across insurance status in younger women showed that compared to privately-insured women, those in other insurance groups had a higher risk of dying, with the highest risk shown in uninsured patients. Of note, younger patients with Medicare insurance were excluded since this group would likely include patients with worse prognosis than those in the older group, complicating the older vs. younger Medicare comparisons. Medicaid/publicly insured patients and uninsured patients had higher mortality compared to those with private insurance regardless of age group although the HRs were higher for younger women. These results suggest that health insurance plays an important role in explaining disparities in breast cancer mortality, as has been noted in the literature [23, 24], and that these disparities are somewhat more pronounced in younger women. Higher mortality in Medicaid patients may be due to challenges with Medicaid insurance processes, whereby patients do not get access to Medicaid insurance until their diagnosis of breast cancer is established. Results of some studies suggest differences in treatment for Medicaid patients compared to privately insured patients [25, 26], with Medicaid patients being less likely to receive more aggressive treatment [26], lessening with age [27], which could be due to older patients qualifying for Medicare. Our results draw similar conclusions to these published reports. When compared to private insurance, patients with Medicaid had higher mortality, with higher risk in younger than older patients. Additional research is needed to disentangle age differences in the relationship of insurance status on breast cancer mortality.

Findings of our study need to be put into context of limitations. Although results are based on population-based data covering the entire state of California, given the scarcity of published data on the age-related differences in mortality, these need further validation in other population-based settings. Further, our survival analyses were adjusted for important clinical characteristics. Importantly, we are unable to account for comorbidities because they are not collected as part of the cancer registry. As such, our findings could be subject to residual confounding from incomplete treatment and comorbidity data in the cancer registry [28], which may be especially relevant when comparing older and younger patients. In addition, although it is likely that very young women (< 40 years) have a higher risk of mortality than older women [3], which could affect the results of our study, excluding this younger group from the analysis had no appreciable effect on the observed mortality measures. We emphasize that due to limited data on this topic, future population-based studies with more detailed treatment, clinical comorbidity data than those available in the registry are needed to validate our findings and potentially explore mechanisms associated with our observed age-related mortality differences.

## Conclusions

Results from our population-based study show that older breast cancer patients have higher risk of dying from breast cancer compared with younger women, with differences more pronounced for NHW, Hispanic, and privately insured women. The prominent Black-White mortality disparity among younger women warrants further study of whether biology, access to treatment, or other factors are driving the particularly poor survival among young non-Hispanic Black women. Health insurance plays an important role in in explaining age-related differences in breast cancer mortality, with greater disparities shown between privately- and non-privately-insured patients in younger than older patients.

## Data Availability

The datasets generated and/or analyzed during the current study are available upon request to the California Cancer Registry [ccrcal.ca.gov].

## Abbreviations

AJCC: American Joint Committee on Cancer
API: Asian/Pacific Islander
CCR: California Cancer Registry
CI: Confidence interval
ER: Estrogen receptor
HER2: Human epidermal growth factor receptor 2
HR: Hazard ratio
ICD-O-3: International Classification of Disease for Oncology, 3rd Edition
NCICC: National Cancer Institute-designated Cancer Centers in California
NHW: Non-Hispanic White
nSES: Neighborhood socioeconomi status
PR: Progesterone receptor
U.S: United States
